# Paediatric Obsessive–Compulsive Disorder and Comorbid Body Dysmorphic Disorder: Clinical Expression and Treatment Response

**DOI:** 10.1007/s10578-022-01314-x

**Published:** 2022-01-20

**Authors:** Jason I. Racz, Sharna L. Mathieu, Matthew L. McKenzie, Lara J. Farrell

**Affiliations:** School of Applied Psychology, Griffith University, Parklands Drive, Southport, QLD 4222 Australia

**Keywords:** Paediatric, Comorbid, Obsessive–compulsive disorder, Body dysmorphic disorder, Presentation

## Abstract

This study explored the expression, occurrence, and treatment outcomes of comorbid body dysmorphic disorder (BDD) in 107 youth (7–17 years) seeking treatment for primary obsessive–compulsive disorder (OCD). In the overall sample, appearance anxiety (AA) was positively associated with OCD-related impairment, severity, symptom frequency, comorbid symptoms, and maladaptive emotion regulation. Comorbid BDD occurred in 9.35% of youth, equally affected males and females, and was associated with older age. AA negligibly reduced following treatment. Compared to those without (a) comorbid BDD and (b) without any comorbidity, youth with comorbid BDD reported greater social impairment and reduced global functioning but did not differ on the occurrence of comorbid anxiety and mood disorders. OCD response or remission rates did not differ. In youth with comorbid BDD, AA did not significantly reduce following treatment. Results suggest a more severe expression accompanies comorbid BDD in youth with OCD, with BDD persisting following OCD treatment.

## Introduction

Approximately 3% of youth experience obsessive–compulsive disorder (OCD) [1], with 25% of lifetime cases emerging by 14 years old [2]. Paediatric OCD is chronic and possesses a high comorbidity rate [3] that often exacerbates its expression, including increased rates of family accommodation [4], as well as OCD severity and is associated with attenuated outcomes [5]. For example, a study by Storch and colleagues [5] indicated that amongst 96 youth with OCD, 74% possessed a comorbidity, which was associated with reduced treatment response and remission following cognitive-behavioural therapy (CBT). Indeed, research indicates that specific comorbidities, such as attention-deficit/hyperactivity disorder, may be associated with a particularly severe and refractory presentation [4, 6].

Notably, OCD and obsessive–compulsive and related disorders, such as body dysmorphic disorder (BDD), co-occur at a greater rate relative to the general population [3]. BDD frequently onsets during adolescence and is defined by an extreme preoccupation with perceived appearance defects, accompanied by repetitive behaviours, and significant distress and/or impairment [2]. Within adult OCD samples, comorbid BDD (OCD + BDD) occurs equally across genders [7–9], however, it may be more likely in older youth given the later adolescent onset of BDD relative to OCD [2]. Importantly, it is reported that such a comorbidity encourages a more complex OCD expression, with studies reporting increased OCD symptom severity [7, 8, 10, 11], earlier age of OCD onset [7, 11], more substantial impairment in functioning [9], and overall greater comorbidity, including anxiety and mood disorders [7, 8, 12]. For instance, in a study of 901 outpatients with OCD (*M*_age_ = 34.4 years), 11.4% experienced comorbid BDD, and these patients tended to be younger, had more severe OCD symptoms, and a higher rate of dysthymia and social phobia [7]. However, to date, there have been no previous studies which have examined the occurrence of BDD exclusively among youth with OCD. This is despite an occurrence of 10.40% in adult samples [13], suggesting a rate more than four times that of population estimates (i.e., 2.20%) [14]. Establishing the occurrence and correlates of this common and severe comorbid presentation in youth with OCD is an important step towards improved outcomes.

Indeed, given the increased complexity and impairment associated with OCD + BDD, it is likely that this presentation may be particularly challenging to treat. Broadly, CBT for the direct treatment of BDD has received empirical support with a meta-analysis of randomised controlled trials (RCT) demonstrating the maintenance of improvements up to four-months after treatment [15]. Studies have highlighted that specific clinical variables associated with adult OCD + BDD (e.g., reduced insight and greater depression) do not appear to reduce effectiveness of CBT for adolescents with BDD [16]. The limited literature examining the effectiveness of treating youth with OCD + BDD suggests improvements across conditions following CBT, however outcomes may be unstable. For instance, one case study reported on exposure and response prevention (ERP) with behavioural activation for an adolescent presenting with BDD and comorbid OCD and major depressive disorder, finding an initial reduction in both OCD and BDD severity; however BDD gains diminished after early treatment cessation [17]. Likewise, an observational longitudinal study including 53 participants aged 12 years or older with BDD and comorbid OCD, reported that whilst improvements in OCD symptoms predicted subsequent comorbid BDD remission, clinical BDD persisted in 50% of participants three months following OCD remission [18]. This possibility, that amongst youth with OCD comorbid BDD may attenuate OCD treatment response, whilst also benefiting to a limited extent from said treatment, warrants further investigation.

### The Present Study

The current study aimed to investigate the clinical expression, occurrence, and treatment outcomes of OCD + BDD in youth with a primary diagnosis of OCD who received intensive CBT for OCD. These aims were achieved by firstly examining associations with BDD symptoms (appearance anxiety) within a sample of youth with OCD. It was hypothesised that appearance anxiety (AA) would be significantly and positively correlated with OCD-related severity, impairment, family accommodation, and symptom frequency; as well as anxiety symptoms, depressive symptoms, externalising symptoms, and maladaptive emotion regulation, and negatively correlated with OCD onset age, insight, adaptive emotion regulation, and global functioning. Further, that AA would decrease significantly from pre-treatment to post-treatment following intensive CBT for youth with OCD.

Secondly, following an exploration of the occurrence of comorbid BDD, we aimed to examine differences across subgroups of youth with either comorbid OCD + BDD, OCD without BDD, or OCD without any comorbidities (OCD-only). The OCD without BDD group was made up of youth without comorbid BDD, with and without other internalising comorbidities. In comparison, youth with OCD and no other comorbidities constituted the OCD-only group. It was hypothesised that compared to youth with OCD without BDD and youth with OCD-only, those with comorbid OCD + BDD would have significantly higher OCD-related severity and impairment, maladaptive emotion regulation, poorer global functioning, and would more likely have comorbid anxiety and mood disorders (relative to youth with OCD without BDD). Further, it was hypothesised that youth with comorbid OCD + BDD would be less likely to be OCD treatment responders and remitters at post-treatment and three-month follow-up relative to other subgroups, although youth with OCD + BDD would report a significant decrease in AA symptoms following intensive CBT for OCD.

## Methods

### Participants

Participants included 107 youth (53.27% female) aged 7 to 17 years (*M*_age_ = 11.94, *SD* = 2.48), with a primary diagnosis of OCD, and their parents, recruited for OCD treatment through community advertising or referral. Youth were recruited for a larger treatment RCT and met the inclusion criteria: primary diagnosis of OCD, parent willing to participate, and stable (i.e., 12 weeks) medication dose before treatment. Exclusion criteria included: the presence of psychosis, intellectual disability, active suicidal ideation, concurrent psychotherapy, or non-proficiency in the English language.

To address subgroup analyses the OCD + BDD clinical subsample (*n* = 10, 50% female, *M*_age_ = 13.80, *SD* = 2.49) was paired with OCD without BDD (*n* = 10, 50% female, *M*_age_ = 13.40, *SD* = 2.17) and OCD-only controls (*n* = 10, 70% female, *M*_age_ = 12.70, *SD* = 2.36) of approximately matched age and gender, via consecutive enrolments into the trial. Youth with autism spectrum disorder or attention-deficit/hyperactivity disorder were excluded from these controls given their more severe OCD clinical expression [4, 19].

### Measures

#### Diagnostic Measures

Anxiety Disorders Interview Schedule for DSM-IV-Parent Version (ADIS-IV-P) [20]. The ADIS-IV-P is a structured parent-interview, assessing child psychological disorders and severity, according to diagnostic criteria [21], and was used to diagnose OCD, and comorbid anxiety, mood, and externalising disorders. Diagnostic decisions were made according to the ADIS-IV-P protocol, based on the reported presentation of symptoms and a severity rating on an 8-point scale. The ADIS-IV-P displays acceptable convergent validity (*r* ≥ .22) with the Children’s Yale-Brown Obsessive–Compulsive Scale symptom checklist [22] and excellent interrater agreement in past analyses of this sample (κ ≥ .84) [19].

Body Dysmorphic Disorder Questionnaire for Adolescents (BDDQ-A) [23]. The BDDQ-A is a four-item dichotomous self-report measure assessing BDD diagnostic criteria [21]. The adult version reports 100% sensitivity and 89% specificity in individuals with a psychiatric diagnosis [23]. Further, in Australian youth the BDDQ-A has demonstrated acceptable reliability (α = .75) [24], and in those reporting high levels of BDD symptoms, known-groups validity [25].

#### Symptom Measures

Appearance Anxiety Inventory (AAI) [26]. The AAI is a 14-item self-report measure of AA on a 5-point Likert scale. A total score of 20 or above represents clinical levels [27]. The AAI has demonstrated convergent validity with the Cosmetic Procedure Screening [26] and excellent internal consistency in the current study (α = .94).

Children’s Yale-Brown Obsessive–Compulsive Scale (CY-BOCS) [28]. The CY-BOCS is a child and parent semi-structured clinical interview of OCD severity rated using 10-items on a 5-point Likert scale across both the Obsession and Compulsion subscales. Symptom insight was measured using one additional item from the CY-BOCS supplementary impairment items. The CY-BOCS displays good interrater reliability (κ = .66) and convergent validity with self-report [28].

Child OCD Impact Scale-Child (COIS-C) [29]. The COIS-C is a 57-item child self-report measure of OCD-related impairment in psychosocial functioning across School, Social, and Home subscales on a 4-point Likert scale. The COIS-C has displayed moderate convergent validity (*r* = .46) with the CY-BOCS symptom checklist [29], and in the current study, excellent internal consistency across the subscales and total (α > .91).

Children’s Global Assessment Scale (CGAS) [30]. The CGAS is a single-item clinician-rated measure of global functioning in youth over the previous two weeks, on a 100-point scale with a score equal to or above 70 indicating normal functioning. The CGAS possesses strong interrater reliability (intraclass correlation coefficient of .73) [31] and good convergent validity with the Child Behaviour Checklist [32].

Obsessive–Compulsive Inventory-Child Version (OCI-CV) [33]. The OCI-CV is a 21-item self-report measure of paediatric OCD symptom frequency on a 3-point Likert scale across the subscales of Washing, Doubting/Checking, Hoarding, Ordering, Obsessing, and Neutralising subscales. The OCI-CV has demonstrated good divergent validity with the Children’s Depression Inventory [33] and, in the current study, acceptable internal consistency across most subscales and in total (α > .78). Whilst the Neutralising subscale (α = .66) demonstrated unstable internal consistency it remained usable considering the exploratory purpose of the current study [34].

Family Accommodation Scale for OCD-Self-Rated Version (FAS-SR) [35]. The FAS-SR is a parent-rated 19-item measure of the frequency and severity of family accommodation of obsessive–compulsive behaviours over the previous month on a 5-point Likert scale. The FAS-SR has displayed good convergent validity with OCD severity [35] and excellent internal consistency in the current study (α = .91).

Multidimensional Anxiety Scale for Children 2nd Edition-Self Report (MASC 2-SR) [36]. The MASC 2-SR is a 50-item child self-report measure of the frequency and severity of anxiety symptoms in youth on a 4-point Likert scale. The MASC 2-SR displays convergent validity with the Beck Youth Inventory Anxiety subscale [37] and the total score possessed excellent internal consistency (α = .92) in the current study.

Child Depressive Inventory 2nd Edition: Self-Report (CDI 2: SR) [38]. The CDI 2: SR is a 28-item child self-report measure of the frequency and severity of depressive symptoms on a 3-point Likert scale. The CDI 2: SR displays discriminant validity in community samples [39]. The total score possessed acceptable internal consistency (α = .87) in the current study.

Child Behaviour Checklist for Ages 6-18 (CBCL/6-18) [40]. The CBCL/6-18 is a 113-item parent-report questionnaire assessing the frequency of behaviours associated with childhood emotional and behavioural disorders, measured on a 3-point Likert scale. The CBCL/6-18 displays convergent validity with various validated measures of psychopathological disorders [41] and excellent internal consistency (α = .91) across the Externalising domain used in the current study.

Cognitive Emotion Regulation Questionnaire (CERQ) [42]. The CERQ is a 36-item self-report measure of cognitive emotional coping strategies in adolescents (i.e., 12 years or older) across adaptive (Acceptance, Positive Refocusing, Refocus on Planning, Positive Reappraisal, and Putting into Perspective) and maladaptive (Self-Blame, Rumination, Catastrophising, and Other-Blame) subscales. For children (i.e., 11 years or younger) different item wording was used (i.e., the CERQ-k) [43]. Items are scored on a 5-point Likert scale. Both adolescent and child versions have demonstrated factorial and criterion validity, respectively [44, 45]. For the current study, the adaptive emotion regulation score had good internal consistency (α > .81). To form a maladaptive emotion regulation score, the four maladaptive subscales were summed with the Expressive Suppression subscale of the Emotion Regulation Questionnaire for Children and Adolescents (ERQ-CA) [46]. The ERQ-CA measures inhibition of emotion-expressive behaviour across four items on a 5-point Likert scale. This maladaptive emotion regulation score had good internal consistency for children and adolescents (α > .74).

### Procedure

Ethical approval for the current procedure was granted by the institution’s human research ethics committee. Participants, including their parents, provided written informed consent. Demographical information was collected and a parent diagnostic interview (i.e., ADIS-IV-P) conducted via telephone, to confirm diagnostic eligibility, which was subject to clinical consensus overseen by a senior clinical psychologist specialising in paediatric OCD (LJF). Ineligible youth were referred to appropriate treatment services, whilst eligible youth completed clinician symptom interviews face-to-face, and questionnaire measures online. Participants completed three intensive three-hour CBT-ERP sessions, over three weeks, with an additional booster session one month later. These sessions were delivered by supervised therapists that were clinically trained postgraduate psychology students undergoing their final year in training or were fully registered. As part of the trial, participants were randomised to a placebo or d-cycloserine augmented condition, which yielded a non-significant time × treatment effect on all primary outcomes [47] and consequently was not controlled for in the current study.

#### Overview of Analyses

To examine hypothesis one, bivariate correlations between AA and clinical characteristics were calculated, using the 82 participants (*M*_age_ = 12.06, *SD* = 2.48) whose AA was assessed at pre-treatment (47.60% male, 9.80% with comorbid BDD). Exclusion of one OCD + BDD participant’s extreme and unreliable parent-report data reduced the sample to 81 for parent-report measures. Given poor normality [48], to control for type one error [49, 50] AA was logarithmically transformed with a conservative effect. To examine subgroup analyses, a series of Kruskal–Wallis ANOVAs assessed OCD severity and impairment between the OCD + BDD, OCD without BDD and OCD-only subsamples. As is common within small clinical samples, adjustments for multiple comparisons often inflate type two error [51], therefore adjustments were not made to alpha rate for multiple comparisons. A series of follow-up Mann–Whitney *U* tests compared the OCD + BDD group to the OCD without BDD and OCD-only groups. To examine treatment response, a reduction in OCD severity of 25% indicated response and a 50% reduction, along with a raw score below 14, signified remission [52].

Given the hypothesis-generating aim and that the data were missing at random, χ^2^(1082) = 1040.63, *p* = .812, item-level missing data were replaced by the participant’s scale mean, and further construct-level missing repeated measures were imputed by carrying the last observation forward to maximise conservative outcomes. Any remaining missing data was excluded pairwise to maximise statistical power [53] and effect sizes were reported regardless of statistical significance as a measure of clinical meaningfulness [54].

## Results

### BDD Symptoms (AA) in Youth with OCD: Clinical Correlates

Correlations between AA (untransformed *M* = 9.54, *SD* = 10.66; transformed *M* = 0.82, *SD* = 0.45) and measures of clinical expression, can be found in Table 1, alongside descriptive statistics. Partially supporting hypothesis one, AA was significantly positively correlated with OCD-related impairment, severity (compulsions and total), and symptom frequency (doubting, ordering, obsessing, neutralising, and total), alongside anxiety and depressive symptoms, and maladaptive emotion regulation.

**Table 1:** Correlations with appearance anxiety and descriptive statistics

Variable (*n*)	*r*	*M*	*SD*
OCD clinical expression			
Age of onset (79)	.14	8.70	3.02
Insight (82)	− .00	1.57	1.01
Impairment (82)	.36**	44.15	30.72
Family accommodation (79)	.06	25.72	17.87
Severity (82)			
Obsessions	.15	13.34	2.42
Compulsions	.24*	13.84	2.31
Total	.23*	27.20	4.06
Symptom frequency (82)			
Washing	.06	3.16	2.26
Doubting	.33**	3.97	2.82
Hoarding	.19	1.81	1.66
Ordering	.31**	2.79	1.70
Obsessing	.25*	3.94	2.19
Neutralising	.32**	1.71	1.64
Total frequency	.41***	17.37	7.26
Comorbidity symptoms			
Anxiety (80)	.32**	72.45	22.34
Depressive (80)	.46***	12.45	7.75
Externalising (79)	.18	8.74	8.44
Emotion regulation			
Adaptive (60)	− .06	52.69	13.84
Maladaptive (58)	.53***	45.29	11.15
Global functioning (78)	.05	53.59	6.79

*OCD* obsessive–compulsive disorder

*p < .05. **p < .01. ***p < .001

### BDD Symptoms (AA) Following OCD Treatment

Supporting hypothesis two, within those who completed AA at pre-treatment (*n* = 82), AA was significantly lower at post-treatment (*M* = 8.05, *SD* = 10.17) relative to pre-treatment (*M* = 9.54, *SD* = 10.66), *t*(81) = 2.56, *p* = .012, *d* = .14.

### The Occurrence of BDD in Youth with OCD

Within the overall treatment-seeking sample (*N* = 107), clinical BDD occurred in 10 individuals at a frequency of 9.35%, 95% confidence interval [3.83, 14.86]. Those with comorbid BDD (*M* = 13.80, *SD* = 2.49) were significantly older than those without BDD (*M* = 11.75, *SD* = 2.41), *t*(105) = − 2.55, *p* = .012, *d* = .85. Gender frequency (male or female) did not differ, *p* = 1.00, ϕ = .02, between participants with (50.0% male) and without BDD (46.4% male).

### Comorbid OCD and BDD: Symptoms, Functioning, and Comorbidity

The Kruskal–Wallis ANOVAs and follow-up Mann Whitney *U* tests assessing OCD severity and impairment between the subgroups are presented in Tables 2 and 3, respectively, alongside descriptive statistics to ease interpretability. Significant omnibus differences emerged on OCD severity (obsessions and total), OCD-related impairment (school, social, and total), adaptive emotion regulation, and global functioning. Partially supporting hypothesis three, the OCD + BDD group ranked significantly higher on OCD-related impairment in the social domain and lower on global functioning than both groups, higher on school and total OCD-related impairment than the OCD-only group, and lower on adaptive emotion regulation and higher on OCD obsession and total OCD severity than the OCD without BDD group.

**Table 2:** Descriptive statistics and Kruskal–Wallis ANOVAs assessing measures of OCD severity and impairment between-groups

Variable	Mean rank, *Mdn* (IQR)			*H*^a^	*n*^b^	η^2^
	OCD + BDD (*n* = 10)	OCD without BDD (*n* = 10)	OCD-only (*n* = 10)			
OCD severity						
Obsessions	20.85, 15.00 (2.00)	10.90, 13.00 (1.50)	14.75, 14.00 (5.25)	6.67*	10, 10, 10	.23
Compulsions	18.95, 15.00 (4.25)	15.40, 14.50 (3.25)	12.15, 14.50 (4.75)	3.05	10, 10, 10	.11
Total	21.25, 30.50 (3.50)	12.00, 27.00 (3.50)	13.25, 29.00 (9.50)	6.58*	10, 10, 10	.23
OCD-related impairment						
School	20.22, 23.00 (17.53)	14.39, 11.00 (17.00)	7.39, 9.00 (9.00)	11.88**	9, 9, 9	.46
Social	19.56, 30.00 (21.00)	11.00, 10.50 (17.50)	9.67, 8.00 (15.50)	8.79*	9, 8, 9	.35
Home	17.44, 28.00 (19.00)	12.88, 19.50 (23.50)	10.11, 14.00 (17.50)	4.23	9, 8, 9	.17
Total	19.00, 81.00 (53.53)	12.31, 40.00 (44.25)	9.06, 30.00 (42.88)	7.89*	9, 8, 9	.32
Emotion regulation						
Adaptive	6.86, 41.00 (18.00)	17.36, 38.00 (24.50)	10.44, 51.00 (10.25)	9.55**	7, 7, 8	.45
Maladaptive	13.43, 49.00 (15.00)	13.57, 44.00 (35.00)	8.00, 38.00 (24.50)	3.66	7, 7, 8	.17
Global functioning	9.70, 50.00 (5.00)	17.80, 52.50 (10.00)	19.00, 55.00 (11.25)	7.17*	10, 10, 10	.25

*df* = 2*.* *OCD* obsessive–compulsive disorder; *IQR* interquartile range; *OCD + BDD* obsessive–compulsive disorder with comorbid body dysmorphic disorder; *OCD-only* obsessive–compulsive disorder without any comorbidities

^a^Kruskal-Wallis test statistic corrected for ties

^b^OCD + BDD, OCD without BDD, and OCD-only pairwise subsample sizes

*p < .05, **p < .01

**Table 3:** Follow-up Mann–Whitney U tests assessing OCD-related impairment between-groups

Variable	Mean rank			*U*	*r*
	OCD + BDD	OCD without BDD	OCD-only		
Obsession OCD severity	14.25	6.75	–	12.50**	.65
	12.10	–	8.90	34.00	.28
Total OCD severity	13.95	7.05	–	15.50**	.59
	12.80	–	8.20	27.00	.39
School OCD-related impairment	11.67	7.33	–	21.00	.41
	13.56	–	5.44	4.00***	.76
Social OCD-related impairment	11.78	5.88	–	11.00*	.58
	12.78	–	6.22	11.00**	.62
Total OCD-related impairment	11.22	6.50	–	16.00	.47
	12.78	–	6.22	11.00**	.61
Adaptive emotion regulation	4.43	10.57	–	3.00**	.74
	6.43	–	9.38	17.00	.33
Global functioning	7.75	13.25	–	22.50*	.49
	7.45	–	13.55	19.50*	.54

Medians, interquartile ranges, and subsample sizes are reported in Table 2. Exact two-tailed significance was reported. *OCD* obsessive–compulsive disorder; *OCD + BDD* obsessive–compulsive disorder with comorbid body dysmorphic disorder; *OCD-only* obsessive–compulsive disorder without any comorbidities; *dash (–)* not compared in analysis

*p < .05. **p < .01. ***p < .001

Fisher’s exact tests assessing comorbidity frequency between groups revealed no significant differences for social anxiety disorder, specific phobia, panic disorder, agoraphobia, generalised anxiety disorder, post-traumatic stress disorder, dysthymia, and major depressive disorder across youth with and without comorbid BDD, failing to support hypothesis four. The comorbid group was higher on the frequency of all disorders, except for dysthymia, where it was lower. Importantly, a trend for greater frequency of comorbid social anxiety disorder was observed (*p* = .057, ϕ = .52) in youth with comorbid BDD (60%) relative to youth without BDD (10%).

### Comorbid OCD and BDD: Treatment Response

Fisher’s exact tests assessing OCD response and remission frequency at post-treatment and three-month follow-up between the groups are presented in Table 4. No significant differences in response or remission frequency emerged at post-treatment or three-month follow-up, opposing hypothesis five.

**Table 4:** Fisher’s exact tests assessing OCD response and remission frequency between-groups

OCD response	Frequency			*p*	*w*
	OCD + BDD	OCD without BDD	OCD-only		
Post-treatment					
Response	40.00%	60.00%	80.00%	.248	.33
Remission	30.00%	40.00%	70.00%	.272	.34
Three-month follow-up					
Response	50.00%	70.00%	80.00%	.500	.27
Remission	40.00%	50.00%	50.00%	1.00	.09

*n* = 30. *OCD + BDD* obsessive–compulsive disorder with comorbid body dysmorphic disorder; *OCD-only* obsessive–compulsive disorder without any comorbidities

Hypothesis six was not supported, as within the OCD + BDD subgroup BDD symptoms (AA) did not rank significantly differently between pre-treatment (*Mdn* = 27.50, *IQR* = 32.00) and post-treatment (*Mdn* = 22.00, *IQR* = 25.25), *T* = 1.00, *n*—ties = 5, *p* = .080, *r* = .44. Specifically, relative to pre-treatment, the AA of four participants ranked lower at post-treatment (sum of ranks = 14.00), whilst only one participant ranked higher (sum of ranks = 1.00), and three participants reported no difference.

## Discussion

This study aimed to explore the clinical expression, occurrence, and treatment outcomes of OCD + BDD in a sample of treatment-seeking youth with a primary diagnosis of OCD. It was broadly hypothesised that BDD symptoms and comorbid BDD diagnoses would be associated with greater severity and impairment, comorbid anxiety and mood disorders, and a reduced response to OCD treatment, whilst BDD symptoms would reduce from pre-treatment to post-treatment.

In the overall sample, hypothesised positive associations between BDD symptoms and OCD-related impairment, severity, and symptom frequency alongside anxiety and depressive symptoms and maladaptive emotion regulation, suggests that even subclinical BDD symptoms may be associated with a more complex and severe paediatric OCD presentation. However, the absence of several predicted associations (i.e., family accommodation, insight etc.) indicate that this may occur only in specific domains or may be worse in older populations [55, 56]. Regarding treatment outcomes, the significant, but not meaningful, reduction in AA from pre-treatment to post-treatment provided partial support for the second hypothesis.

Nearly one in 10 youth with OCD experienced comorbid BDD in the current sample, much greater than previous estimates in the general adolescent population [14]. Youth with OCD + BDD were older than those without BDD, but gender frequency did not differ. Amongst these comorbid youth, subgroup analyses partially supported hypothesis three, providing preliminary evidence of the association between this comorbidity and impairment in paediatric OCD. Specifically, BDD comorbid youth reported higher OCD-related impairment within social contexts and reduced levels of global functioning, beyond that accounted for by comorbidity alone. This is concerning, given the lifelong impact of disruptions to psychosocial development [57]. Moreover, greater OCD obsession and total severity, alongside lower adaptive emotion regulation, relative to those without BDD suggests the similarities between these obsessive–compulsive and related disorders may encourage a more severe presentation. Whilst the absence of increases in mood or anxiety disorders in youth with OCD + BDD fails to support hypothesis four, meaningful effects suggest a trend toward greater overall comorbidity. In particular, social anxiety disorder occurred at a higher rate in such individuals (i.e., 60%) relative to those with OCD without BDD (i.e., 10%), suggesting such findings in adults with OCD + BDD [7, 8] may be present in youth. Similarly, the OCD + BDD group did not significantly differ on OCD response or remission, opposing hypothesis five but mirroring findings from adult inpatients [11]. However, meaningful differences signify a trend toward poorer initial response for youth with OCD + BDD (i.e., 40%) relative to those without comorbid BDD (i.e., 60%) and no comorbidities (i.e., 80%). Both these trends beckon further investigation in an adequately powered analysis, to clarify the effect comorbid BDD has on overall comorbidity and treatment outcomes. Finally, failing to support hypothesis six, the OCD + BDD group did not report a significant reduction in AA following treatment, with most continuing to report clinical levels at post-treatment, supporting findings that BDD symptoms largely persist when OCD reduces in comorbid patients [18]. Together with no meaningful reduction in the overall sample, these results emphasise that this comorbid presentation warrants a modularised condition-specific treatment to lead to meaningful BDD improvements [58].

### Limitations and Future Research

Despite this study’s large sample of youth with paediatric OCD and analyses across comorbid control groups allowing the unique effects of comorbid BDD to be disentangled from overall comorbidity, several limitations exist. The lack of a primary BDD without comorbidity control made disentangling the additive effect of BDD, from that of a unique OCD + BDD expression, impossible. Secondly, small exploratory subsamples likely resulted in several meaningful differences failing to reach significance. Finally, given the exploratory aim, carrying last observations forward for missing data aimed to estimate outcomes conservatively, however, given attrition often increases alongside severity, generalisability may be limited for severe presentations [59]. Nevertheless, these results extend several adult findings into youth, providing a hypothesis-generating framework for exploration in adequately powered samples. Future studies should consider controlling for the additive effects of BDD, as doing so would improve our understanding of the unique effects OCD + BDD comorbidity have on clinical expression and treatment outcomes.

### Conclusions and Implications

As one of the first explorations of comorbid BDD in paediatric OCD, these findings reinforce that practitioners should remain aware that the often hidden [60] and underdiagnosed [61] BDD appears relatively common within paediatric OCD and is associated with worse impairment. Furthermore, as BDD symptoms appear to persist following OCD treatment, practitioners should consider using a unique CBT protocol for its treatment in comorbid youth.

## Summary

The clinical expression, occurrence, and treatment outcomes of comorbid BDD were explored in a large, treatment-seeking sample of youth with a primary diagnosis of OCD. Participants (*N* = 107) aged 7–17 years (*M*_age_ = 11.94, 46.70% male) were recruited for OCD treatment as part of a larger randomised-controlled trial. Study measures were completed at pre-treatment, post-treatment, and three-month follow-up. Among the 107 youth with OCD, greater AA was significantly associated with greater OCD-related impairment, severity, symptom frequency, anxious and depressive symptoms, and maladaptive emotion regulation. Comorbid BDD occurred in 9.35% of youth with OCD, equally affecting both males and females. Moreover, those with comorbid BDD were older than those without. AA significantly reduced following OCD treatment, although this difference was negligible in size. For subgroup analyses, youth with comorbid BDD were compared to two age and gender matched subsamples of youth, including those (a) without comorbid BDD and (b) without any comorbidity. Youth with comorbid BDD reported greater OCD-related impairment in social settings and reduced global functioning relative to those with and without other comorbidities but did not significantly differ from those without comorbid BDD on the occurrence of comorbid anxiety and mood disorders. OCD response or remission rate did not differ across the subsamples with and without comorbid BDD at post-treatment or three-month follow-up. Furthermore, youth with comorbid BDD did not report a significant reduction in body dysmorphic symptoms following OCD treatment. This study suggests comorbid BDD is common in youth with OCD, relative to previous adolescent general population estimates, is accompanied by a unique and more severe clinical expression, and that BDD symptoms appear to persist following the treatment of OCD.
